# Catalytic water dissociation by greigite Fe_3_S_4_ surfaces: density functional theory study

**DOI:** 10.1098/rspa.2016.0080

**Published:** 2016-04

**Authors:** A. Roldan, N. H. de Leeuw

**Affiliations:** 1 School of Chemistry, Cardiff University, Main Building, Park Place, Cardiff CF10 3AT, UK; 2 Department of Earth Sciences, Utrecht University, Princetonplein 9, Utrecht 3584 CC, The Netherlands

**Keywords:** iron sulfide, magnetite, solid–water interface, synergistic adsorption, electronic structure

## Abstract

The iron sulfide mineral greigite, Fe_3_S_4_, has shown promising capability as a hydrogenating catalyst, in particular in the reduction of carbon dioxide to produce small organic molecules under mild conditions. We employed density functional theory calculations to investigate the {001},{011} and {111} surfaces of this iron thiospinel material, as well as the production of hydrogen ad-atoms from the dissociation of water molecules on the surfaces. We systematically analysed the adsorption geometries and the electronic structure of both bare and hydroxylated surfaces. The sulfide surfaces presented a higher flexibility than the isomorphic oxide magnetite, Fe_3_O_4_, allowing perpendicular movement of the cations above or below the top atomic sulfur layer. We considered both molecular and dissociative water adsorption processes, and have shown that molecular adsorption is the predominant state on these surfaces from both a thermodynamic and kinetic point of view. We considered a second molecule of water which stabilizes the system mainly by H-bonds, although the dissociation process remains thermodynamically unfavourable. We noted, however, synergistic adsorption effects on the Fe_3_S_4_{001} owing to the presence of hydroxyl groups. We concluded that, in contrast to Fe_3_O_4_, molecular adsorption of water is clearly preferred on greigite surfaces.

## 1. Introduction

The necessity of mitigating climate change has led to policies to regulate and minimize the concentration of carbon dioxide in the atmosphere [1]. As such, much research is dedicated to the search for new materials built from abundant compounds which are capable of using CO_2_ as a feedstock for fuels and valuable compounds [2–6]. Reduction reactions involving CO_2_ are challenging because they require energy to generate reduced forms [7]; in particular, the production of CO is considered an important objective in the context of producing renewable carbon feedstock chemicals [8]. Recent attention has focused on the photo- and electro-catalysis of CO_2_ to fuels via synthesis gas (syngas) [3,9–11], although part of the challenge lies in obtaining hydrogen as a reducing agent from an abundant source, i.e. water [12–14].

Understanding the properties of a catalyst in contact with water is of crucial importance, not only for H_2_ generation but also in many areas of fundamental research and applications [15]. Apart from the direct role of water in many (photo-) catalytic surface reactions, water–surface interactions play an intrinsic role in understanding wetting and corrosion, and in the description of electrochemical interfaces. For example, the electro-reduction of CO_2_ catalysed by greigite, an iron sulfide that is isomorphic with magnetite, takes place in wet conditions using water as a hydrogen-donating species [16]. A major objective in studying water–surface interactions, where greigite is no exception, is to determine whether water is adsorbed molecularly or dissociatively. The chemical properties of the water dissociation products (OH, H and O) are very different from those of the water molecule and may lead, for example, to the surface and bulk oxidation of many materials [17]. The occurrence or not of H_2_O dissociation has significant implications for many chemical processes, e.g. the CO_2_ reduction already mentioned, and also reveals much about the reactivity of a surface towards other chemical species [18–20]. Molecular H_2_O can be used to probe site-specific structure–reactivity relationships, especially on oxides and semiconductors, but to distinguish H_2_O from OH is complicated owing to the similarities in many of the properties of these two species. Identifying irreversible water dissociation is considerably easier than identifying reversible water dissociation, because the former is usually accompanied by modification of the host substrate (e.g. oxide formation) [15].

Understanding processes such as water sorption and dissociation is important for the development of efficient catalysts [21]. Although still lacking for sulfides, there is an extensive literature on water–metal [15,17] and water–oxide interphases [22], describing processes such as surface stability [23–29] and preferred direction for surface growth of minerals [30–33]. Electrochemical reduction under wet conditions may lead to H_2_ evolution on greigite surfaces, as has been shown on other sulfides, e.g. Mo_3_S_4_ [34]. On Fe_3_O_4_, the oxide isomorph of greigite [35], different adsorption modes have been identified upon its interaction with water: dissociative chemisorption and, at higher coverage, physisorbed H_2_O in a condensed ice conformation [36,37], in agreement with molecular dynamics models [32,38]. Density functional theory (DFT) methods have also reported exothermic molecular and dissociative adsorption of one H_2_O on an Fe-terminated Fe_3_O_4_(111) surface and a hydrogen-bonded second water molecule, which, via its oxygen, forms a hydronium ion-like structure [39–41]. Hence, water molecules provide not only a reaction environment but also hydrogen, hydroxy and/or oxygen subspecies that can also react further at the catalyst interface.

We studied various adsorption modes of a single H_2_O molecule on a number of low Miller index surfaces of greigite, followed by its effect on the adsorption of a second water molecule. Although the adsorption of water molecules does not influence the reconstruction of the surface for either metals or ionic solids [17], the effect of water on surface relaxation can be significant. For example, theoretical studies have predicted that the inward relaxation of metal cations on basal plane materials can be restored by water adsorption [17]. We systematically discuss the effect of adsorption/dissociation of H_2_O on the surfaces and its implications for the electronic structure, while also identifying a process of synergistic co-adsorption.

## 2. Computational details

Greigite (Fe_3_S_4_) is an inverse spinel-structured sulfide mineral analogue to the magnetite [35], whose structure contains two kind of Fe atoms: octahedral (Fe_B_) and tetrahedral (Fe_A_) [35,42]. The high-spin alignment is antiparallel, leading to a half metallic character, owing to the presence of Fe(II) in the octahedral sites [43].

### (a) Density functional theory calculations

We carried out a systematic DFT-D2 study of the greigite {001}, {011} and {111} surfaces and their interaction with water. All calculations were performed using the Vienna Ab initio Simulation Package [44,45], where the ion–electron interactions were represented by the projector-augmented wave method [46] and the electron-exchange correlation by the generalized gradient approximation (GGA) with the Perdew–Wang functional (PW91) [47], employing the spin interpolation formula of Vosko *et al*. [48]. All the calculations include the long-range dispersion correction approach by Grimme [49], which is an improvement on pure DFT when considering large polarizable atoms [21,50–54]. We used the global scaling factor parameter optimized for PBE (*s*_6_=0.75). The Kohn–Sham valence states were expanded in a plane-wave basis set with a cut-off at 600 eV for the kinetic energy [55]. This high value for the cut-off energy ensured that no Pulay stresses occurred within the cell during relaxations. The initial magnetic moments were described with high-spin distributions in both types of Fe, i.e. octahedral (B) and tetrahedral (A) Fe in the Fe_A_(Fe_B_)_2_S_4_ spinel structure, by a ferrimagnetic orientation [35]. We employed Monkhorst–Pack grids of 4×4×1 K-points for Fe_3_S_4_{001} and 5×5×1 K-points for Fe_3_S_4_{011} and Fe_3_S_4_{111}, which ensures electronic and ionic convergence [56].

We used the Hubbard-like approximation (*U*) for an accurate treatment of the electron correlation in the localized *d*-Fe orbital of this transition metal [57,58]. This improves the description of localized states in this type of system where standard local density approximation and GGA functionals fail [59]. A problem with this approximation is the rather empirical nature of the U parameter choice, a feature that also appears when using hybrid functionals, because the amount of Fock exchange is system dependent [59–62]. Therefore, we followed the approach used by Devey *et al*. [63] (*U*_eff_=1 eV), the reliability of which has been tested for catalytic processes [16,64]. The geometries of all stationary points were obtained with the conjugate-gradient algorithm and considered converged when the force on each ion dropped below 0.02 eV Å^−1^, whereas the energy threshold, defining self-consistency of the electron density, was set to 10^−5^ eV. In order to improve the convergence of the Brillouin-zone integrations, the partial occupancies were determined using the tetrahedron method with Blöchl correction smearing, with a set width for all calculations of 0.02 eV. These smearing techniques can be considered as a form of finite-temperature DFT, where the varied quantity is the electronic free energy [55]. Finally, we increased the numeric accuracy and the self-consistent threshold up to 10^−7^ eV to obtain a more accurate electronic structure.

### (b) Slab model

The Fe_3_S_4_ surfaces were prepared by cutting the bulk structure and creating slab models using the METADISE code [65]. This code not only considers periodicity in the plane direction, but also provides different atomic layer stacking, resulting in a null dipole moment perpendicular to the surface plane [66]. We considered the three surfaces {001},{011} and {111}, with respective surface areas of 81.0, 132.3 and 93.5 Å [2], as well as their different terminations. Each slab contains 56 atoms (24 Fe and 32 S) per unit cell, and we added a vacuum width of 12 Åbetween periodic slabs, which is big enough to avoid interactions between the slab and its images. The slabs are also thick enough to relax the two uppermost Fe_3_S_4_ layers until energy convergence, keeping the bottom layer frozen to model the bulk structure. To obtain the properties of an isolated H_2_O molecule, we placed it in the centre of a 15×16×17 Å[3] simulation cell to avoid lateral interactions, with broken symmetry, and using the same criteria of convergence as for the iron sulfide slabs.

### (c) Slab characterization

We describe the atomic charges and derive magnetic moment by means of a Bader analysis [67,68], where the electron (and spin) density associated with each atom is integrated over the Bader volume of the atom in question, as implemented in the Henkelman algorithm [69]. Thus, owing to the changes in the effective atomic radii with the oxidation state of the ion, the Bader volume is not calculated as a sphere of constant radius but is charge density dependent. Even so, the electron delocalization of the DFT method leads to an underestimation of atomic charges, i.e. the DFT-derived charges are smaller than the formal oxidation states. However, they can be used effectively in a direct comparison and to monitor changes in charges, for example as an effect of surface adsorption.

In addition to the steady states of reactants and products, we searched for the saddle point linking both systems. This saddle point is the reaction transition state (TS) and determines the kinetics of the process. We identified the TSs by means of the dimer method [70,71], which searches the TS by giving an initial atomic velocity towards the particular final state (product(s)). From an initial configuration, we generated the initial velocities by making two equal and opposite small finite-difference displacements in the coordinates of the reactant molecule. The method then finds a nearby saddle point by rotation and translation steps implemented with a conjugate-gradient optimizer. We further confirmed the identified saddle point (TS) by a vibrational frequency calculation, in which only one imaginary frequency is obtained corresponding to the reaction coordinate. Afterwards, the dimer images were relaxed to the neighbouring local minima. In a successful search, one of the images will minimize into the initial state, and the other will become the final state.

We calculated the adsorption energies (*E*_ads_) per molecule on the Fe_3_S_4_ surfaces via equation (2.1) and via equation (2.2) for the interaction of a second molecule with the surface (Δ*E*),

2.1 $$E_{\mathrm{ads}}=\frac{E_{H_{2}O\;:\;{\mathrm{Fe}}_{3}S_{4}}-\left(E_{{\mathrm{Fe}}_{3}S_{4}}+n\cdot E_{H_{2}O}\right)}{n}$$

and

2.2 $$\Delta E=E_{2\cdot H_{2}O\;:\;{\mathrm{Fe}}_{3}S_{4}}-\left(E_{H_{2}O\;:\;{\mathrm{Fe}}_{3}S_{4}}+E_{H_{2}O}+E_{\mathrm{coh}}\right),$$

where *E*_H_2_O : Fe_3_S_4__ is the total energy of a molecule interacting with the Fe_3_S_4_ slab (two molecules in the case of 2⋅H_2_O:Fe_3_S_4_), *E*_Fe_3_S_4__ is the energy of the naked Fe_3_S_4_ slab and *E*_H_2_O_ is the energy of an isolated molecule in vacuum. When there is more than one molecule in the system, we subtracted the interaction energy between the multiple molecules (*E*_coh_) to isolate its contribution to *E*_ads_. We derived the cohesive energy (*E*_coh_) between molecules by equation (2.3) where the energy (E_2⋅H_2_O_) is the water without the slab, but maintaining the same configuration as in the co-adsorbed situation.

2.3 $$E_{\mathrm{coh}}=\frac{E_{2\cdot H_{2}O}-2\cdot E_{H_{2}O}}{2}.$$

Upon adsorption, we calculated the positive or negative stabilization (deformation) energy (*E*_def_) of the surface (single-point energy, $E_{\mathrm{surf}}^{\mathrm{Mol}\;:\;{\mathrm{Fe}}_{3}S_{4}})$ with respect to the bare slab (single-point energy, $E_{\mathrm{surf}}^{{\mathrm{Fe}}_{3}S_{4}})$ via equation (2.4). *E*_def_ helps us to quantify the distortion of the surface affected by the water molecule(s),

2.4 $$E_{\mathrm{def}}=E_{\mathrm{surf}}^{\mathrm{Mol}\;{:\mathrm{Fe}}_{3}S_{4}}-E_{\mathrm{surf}}^{{\mathrm{Fe}}_{3}S_{4}}.$$

We also defined the energy barrier (ΔE^TS^) for the dissociation process as the difference between the initial state (adsorbed molecule) and the TS, equation (2.5), and the reaction energy (*E*_R_) as the total energy difference between the final state (products) and the initial state (reactants), equation (2.6),

2.5 $$E_{A}=E_{\mathrm{TS}}-E_{\mathrm{initial}}$$

and

2.6 $$E_{R}=E_{\mathrm{final}}-E_{\mathrm{initial}}.$$

We plotted the charge density flux with a positive and negative contour of the electron density difference as Δ*ρ*=*ρ*_H_2_O : Fe_3_S_4__−(*ρ*_Fe_3_S_4__+*ρ*_H_2_O_), where *ρ* is obtained from a single-point calculation. This shows the electron density reallocation upon the deposition and interaction of H_2_O, which is in good agreement with the electronic analysis obtained from the density of states and the charges.

## 3. Results and discussion

### (a) Greigite surfaces

**Figure 1.**
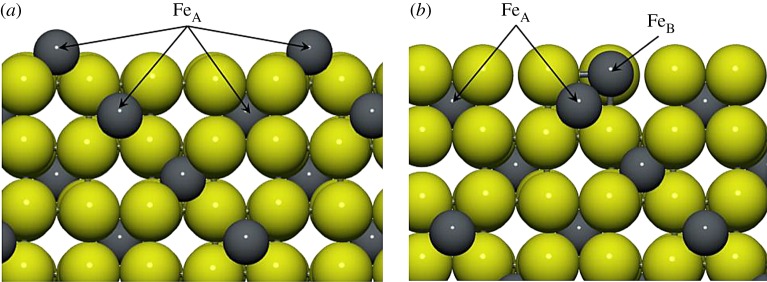
Cross-sectionalview of the unrelaxed Fe_3_S_4_{001} surfaces: termination-A (*a*) and -B (*b*). Arrows show the Fe_A_ above and below the top S layer. Colour scheme: dark grey represents Fe and yellow is S. (Online version in colour.)

We modelled three low Miller index surfaces by cutting the Fe_3_S_4_ bulk crystal to obtain different non-dipolar atomic terminations. The cubic bulk (Fe_A_(Fe_B_)_2_S_4_) unit cell symmetry in the *a*-, *b*- and *c*-axis makes the (001), (010) and (100) surfaces equivalent as well as the (011), (101) and (110) surfaces. Owing to the presence of non-equivalent Fe ions, each surface has two distinct terminations, depending on the relative Fe_A_ position with respect to the uppermost sulfur layer before atomic relaxation (termination -A or -B). For instance, figure 1 shows a schematic representation of both terminations of the Fe_3_S_4_{001} before relaxation; see the electronic supplementary material, figure S1, for all surfaces studied.

**Figure 2.**
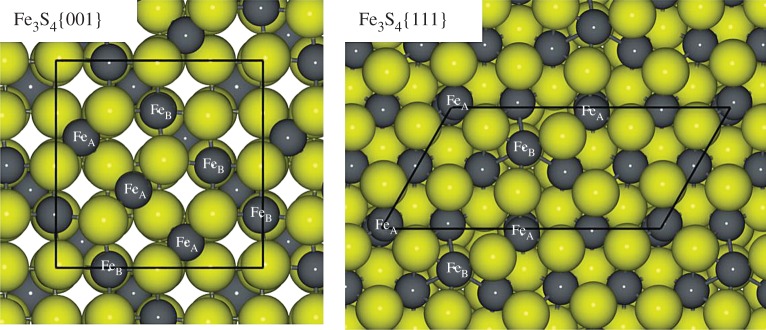
Orthographic top view of {001} and {111} surfaces present on solvothermal synthetized nanoparticles(inset black frame shows the unit cell) [16]. Colour scheme: Fe and S are represented by dark grey and yellow, respectively, balls and sticks. (Online version in colour.)

The simulation slabs of the {001} and {111} surfaces were symmetrical along the *z*-axis, but the {011} slab, where top and bottom layers are complementary, is asymmetrical. In this case, the cleavage energy is actually related to the energy to create both top and bottom surfaces of the slab but we still used this average surface energy for both terminations as we were primarily interested in a comparison between pure and hydrated surfaces. Before relaxation, the order of increasing surface energies is {001}-A <{111}-A <{011}<{111}-B < (001)-B, which remains the same on relaxation. We have summarized the surface and stabilization energies in table 1 and the electronic supplementary material. Surface {001}-A is the lowest-energy surface, both before and after relaxation, followed by the {111} A-termination and then the {011}. These surface energies are comparable with those of Fe_3_O_4_ [72–74] and FeNi_2_S_4_ [21] (table 1). In agreement with the computed surface energies, solvothermal synthetized Fe_3_S_4_ nanocrystal expressed {001} and {111} surfaces (figure 2) [16].

**Table 1.** Surface energy (*γ*) values in J m^−2^ of the most stable terminations after structural relaxation of the prominent Fe_3_S_4_, Fe_3_O_4_ [72] and FeNi_2_S_4_ [21].

	*γ*_*r*_-Fe_3_S_4_	*γ*_*r*_-Fe_3_O_4_	*γ*_*r*_-FeNi_2_S_4_
{001}	0.6	1.0	0.2
{011}	1.0	1.4	1.5
{111}	0.8	1.1	1.3

The spin moments are sensitive to structural changes, showing a spin variation on the two types of Fe, which leads to a different magnetization of saturation per formula unit compared with a bulk analysis (*m*_s_(Fe_A_)=2.8 *μ*_*B*_ and *m*_s_(Fe_B_)=3.0 *μ*_*B*_), whereas the total magnetization is 3.44 *μ*_*B*_ [35]. In the {001} surface, spins on both Fe increase slightly by 0.1 *μ*_*B*_; in the {011} surface, the Fe remain as in the bulk structure; but in the {111} surface, the Fe spin orientation changes, thereby decreasing *M*_s_. These variations are generated because the spins on external atomic layers can be inclined randomly at various angles with respect to the direction of the net moment, thereby modifying the total magnetic moment [75].

Next, we obtained an overview of the surface electronic structure by integrating the local density of states from the Fermi level to a bias by using the Tersoff–Hamann formalism [76], which is expressed as scanning tunnelling microscopy (STM) images implemented in their most basic formulation, approximating the STM tip by an infinitely small point source. STM at constant current mode follows a surface of constant integrated local density of states [50]. Although there are currently no experimental STM measurements in the literature, we have provided STM models in the electronic supplementary material, figure S2, which resemble those for Fe_3_O_4_ [72], and the surfaces shown by high-resolution transition electron microscopy obtained from pure greigite nanoparticles [16]. On the Fe_3_S_4_{001} STM, one can see the parallel thick lines corresponding to the sulfur (bright areas) and the Fe_A_ row at lower density. We found a similar arrangement on the Fe_3_S_4_{011}. Finally, the simulated STM of the Fe_3_S_4_{111} shows the maximum current intensity above the Fe_B_, embedded in lower-density S atoms, where the darker areas correspond to Fe_A_ sites. We also derived the Wulff morphology from the relative surface stabilities [77] (electronic supplementary material, figure S3), where the Fe_3_S_4_ morphology in vacuum and under thermodynamic equilibrium is a cubic crystal with edges truncated by {111} and {011} planes.

### (b) Adsorption of a H_2_O on Fe_3_S_4_

**Figure 3.**
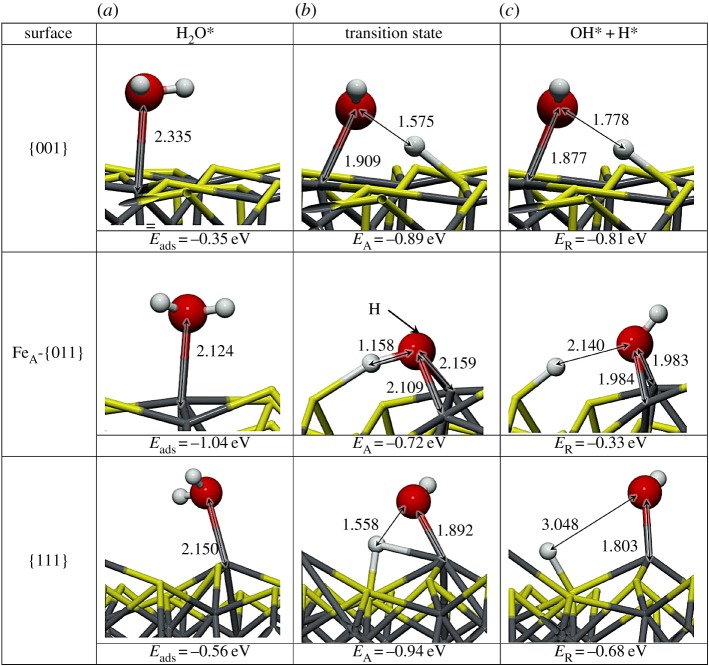
Schematic representation of reactant (*a*), transition state (*b*) and products (*c*) on the H_2_O dissociative process on the {001}, {011} and {111} Fe_3_S_4_ surfaces. Grey balls depict Fe, yellow depicts S, red is O and white balls represent H. The arrows indicate the position of the hidden hydrogen and all distances are in Å. (Online version in colour.)

We focused on the most stable surface terminations (-A) to study their behaviour towards H_2_O adsorption and dissociation. We placed an H_2_O molecule on several non-equivalent positions on each surface and allowed both the surface and the molecule to relax without any restrictions. From all these optimizations (almost 40), we present the most favourable adsorption configurations in figure 3 and summarize their adsorption properties in table 2. We also studied the thermodynamics and kinetics of the H_2_O dissociation process, which may take place after or during adsorption of the molecule onto the surface. The viability of the dissociation is described by means of the reaction energy (*E*_*R*_), whereby an endothermic value of *E*_*R*_ indicates an unlikely dissociative process.

**Table 2.** Adsorption energy with (and without) the dispersion correction (*E*_ads_), distances (*d*), angle (<) and changes in Fe site spin density (Δ*m*_s_) upon H_2_O molecule adsorption on the most stable site on Fe_3_S_4_{001},{011} and {111} surfaces. The gas phase H_2_O molecule has *d*_O–H_=0.971 Å and <_H–O–H_ =104.7°. Note that Fe_A_ is oriented antiparallel (negative sign) with the magnetic field.

site	*E*_ads_ (eV)	*d*_*O*−*Fe*_ (Å)	*d*_O–H_ (Å)	<_H–O–H_ (°)	Δ*m*_s_(Fe) (*μ*B)
Fe_B_-(001)	−0.35 (−0.19)	2.335	0.977	105.3	−0.2
Fe_A_-(011)	−1.04 (−0.85)	2.124	0.976	106.6	0.0
Fe_B_-(011)	−1.11 (−1.02)	2.048	0.972	109.2	0.0
Fe_B_-(111)	−0.56 (−0.43)	2.150	0.977	106.1	−0.4

**Figure 4.**
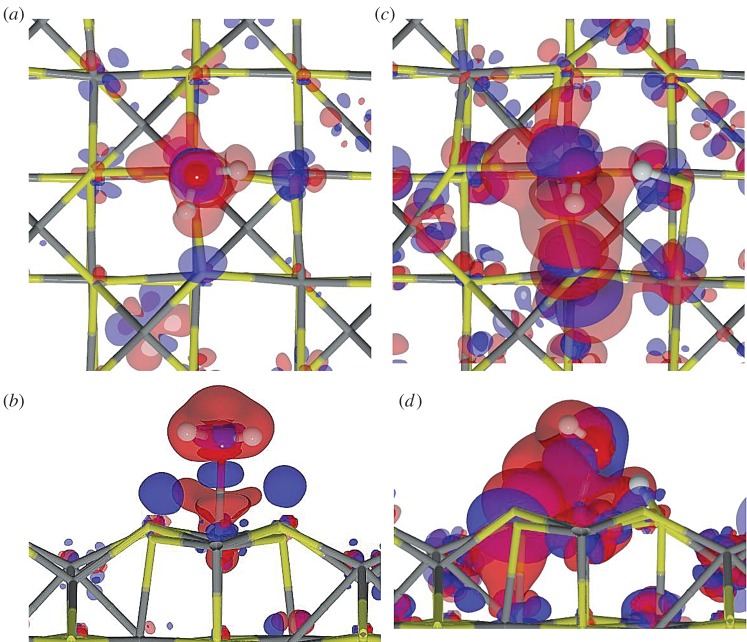
Charge density flow diagrams for (*a*,*b*) molecular and (*c*,*d*) dissociative adsorption of an H_2_O onFe_3_S_4_{001}, where red clouds show depletion and blue gain of charge density (isovalue is ±0.01 *e*^−^ Å^−3^). Grey and yellow sticks depict Fe and S, respectively; red and white balls represent O and H, respectively. (Online version in colour.)

Fe_3_S_4_{001}. The adsorption of an H_2_O on the {001} surface takes place exclusively on one of the four Fe_B_ present at the surface (coverage of 0.25 ML), whereas the S layer repels the lone electron pairs of oxygen, thereby blocking the attractive interaction with the Fe_A_ underneath. On this surface, the molecule lies with its oxygen at 2.335 Åfrom the Fe_B_ with the H pointing towards neighbouring S atoms, which stretches the H_2_O angle. The molecule's adsorption energy is −0.35 eV. The Fe_B_ site moves towards the molecule, rising 0.161 Åfrom the surface plane, which rearranges its orbitals and modifies the spin density by 0.2 *μ*_*B*_. In addition to the Fe_B_ movement, the Fe_A_ moves into the bulk by 0.04 Å, although the surface energy only changes by 0.10 eV. Upon water adsorption, we analysed the molecule–surface interaction using the charge flow diagram in figure 4*a*. Although the charge transfer is negligible, we found a large charge density accumulation between H_2_O and the surface (blue shadow under the O atom in figure 4*a*,*b*). The formation of this new bond is mostly owing to the electronic contribution from Fe_B_ and the molecule (red clouds). Note also the S-polarization towards the H atoms (distance S…H is 2.748 Å), as well as the electron rearrangement in nearby Fe_A_, which modifies its magnetic moment to 0.1 *μ*_*B*_.

Once the molecule was adsorbed on the Fe_3_S_4_{001}, we stretched an H towards a polarized surface S, leading to both thiol (–SH) and hydroxy (–OH) groups on the surface, but this configuration is 0.46 eV higher in energy than H_2_O in the gas phase, indicating that molecular water adsorption is preferred over dissociation on the Fe_3_S_4_{001}. Upon dissociation, the Fe–O distance shrinks by 0.46 Å, whereas the S from the –SH group rises in the surface by almost 0.08 Å. The O–H scission takes place through a late TS with an energy barrier of 0.89 eV, making the process both thermodynamically and kinetically unfavourable. Despite the oxidizing character of the –OH groups (q(OH)=−0.7 *e*^−^), the metal does not get oxidized, the electrons come mostly from the Fe–S where the H is located. The charge density rearrangement is shown in figure 4*c*,*d*, which indicates the charge density of the dissociated H building a bond of covalent character with S, and re-allocation of the density along the Fe–O bond. This distortion of the electronic structure leads to a synergistic effect upon co-adsorption of a second molecule on neighbouring sites [21]. We have summarized the dissociative adsorption characteristics in table 3.

**Table 3.** Reaction energies (*E*_*R*_) with respect to isolated systems, distances (*d*) and OH charge (*q*(OH)) on the (001), (011) and (111) Fe_3_S_4_ surfaces. Figure 3 shows the schematic of reactants, TSs and products.

site	*E*_*R*_ (eV)	*d*_*O*–*Fe*_ (Å)	*d*_O–H_ (Å)	*d*_O⋯H_ (Å)	*d*_S–H_ (Å)	*q*(OH) (e^−^)
Fe_B_-(001)	0.81	1.877	0.976	1.778	1.393	−0.7
Fe_A_-(011)	0.33	1.984	0.975	2.140	1.354	−0.8
Fe_B_-(011)	0.39	1.857	0.974	1.617	1.406	−0.7
Fe_B_-(111)	0.68	1.803	0.973	3.048	1.355	−0.7

**Figure 5.**
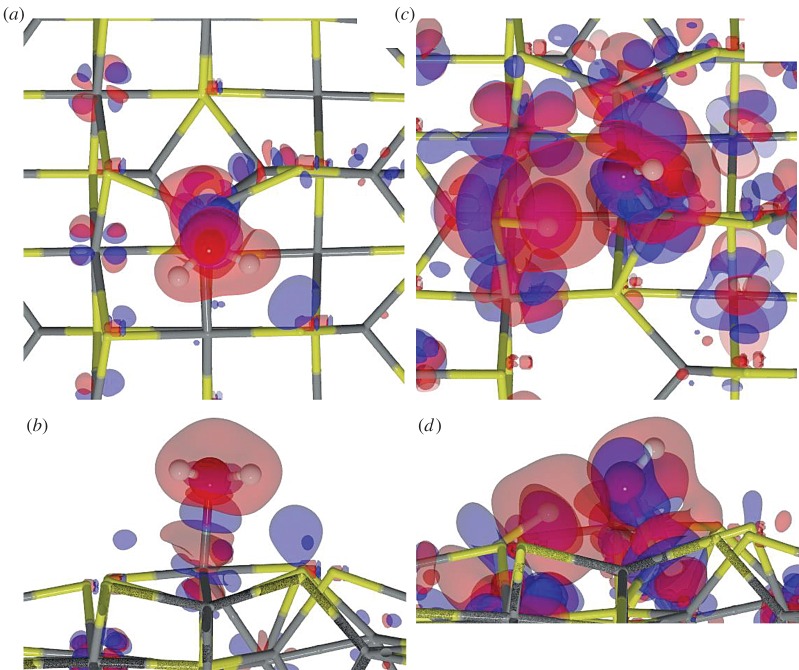
Charge flow diagrams for (*a*,*b*) molecularand (*c*,*d*) dissociative adsorption of H_2_O on Fe_A_ of the Fe_3_S_4_{011}. Red clouds show depletion and blue gain of charge density (isovalue is ±0.01 *e*^−^ Å^−3^). Grey and yellow sticks depict Fe and S, respectively; red and white balls represent O and H, respectively. (Online version in colour.)

Fe_3_S_4_{011}. Even though the Fe_3_S_4_{011} surfaces do not appear in the solvothermal synthetized nanoparticles, we calculated this surface affinity towards H_2_O adsorption because it may still exist in samples derived from other preparation methods. This surface contains more low-coordinated Fe_A_ and Fe_B_ than the {001} and {111} surfaces, which enhances the interaction with H_2_O molecules; *E*_ads_∼1 eV at a low coverage of 0.17 ML. The oxygen–metal distance is slightly shorter than on the other surfaces, in agreement with the stronger adsorption energy, owing to the lower coordination of the metals. The bond between H_2_O and Fe_B_ generates a strong relaxation of the surface, which moves Fe_B_ outwards by up to 2.1 Å, displacing the surrounding S atoms and leaving a distance S…H of only 2.061 Å. This rearrangement is reflected in the surface deformation energy of 0.14 eV per cell. However, when H_2_O adsorbs on Fe_A_, the metal centre only rises by 0.060 Å (*E*_def_=0.1 eV), and the molecule remains almost parallel to the surface. Upon H_2_O adsorption, the charge transfer and the change in spin density is negligible in both metallic sites, which agrees well with the surface deformation. The electronic rearrangement owing to the H_2_O adsorption on Fe_A_ is shown in figure 5*a*,*b*, indicating the bond between the metal centre and the molecule and the S polarization towards the molecule's H.

The dissociation of H_2_O on the Fe_3_S_4_{011} is expected to be more favourable than on the {001} and {111} owing to the large number of dangling bonds at the surface. We deprotonated the water molecule by elongating the H–O bond until the H was placed on the strongly polarized S site. The OH group, which was originally on Fe_A_, then bridged both Fe_A_ and Fe_B_ at distances of 1.983 and 1.984 Å, respectively (*E*_def_=1.31 eV). The dissociation process from the adsorbed molecule on Fe_A_ has an energetic barrier of 0.72 eV. The adsorption energy of the [OH+H] is 0.71 eV below the gas phase molecule, showing it to be thermodynamically plausible. However, it is still above the molecular adsorption energy by 0.33 eV, making the dissociation unlikely either on Fe_A_ or on Fe_B_ (*E*_*R*_=0.34 eV). The electron density distribution, in figure 5*c*,*d*, shows the formation of an S–H covalent bond, with a 0.5 *e*^−^ transfer coming mostly from the sulfur and 0.1 *e*^−^ from the transferred H. It also shows a charge transfer of 0.8 *e*^−^ to the OH group mostly from Fe_A_. One can also distinguish the electron lone pair localization at both sides on the oxygen atoms and the density rearrangement on the metallic centres nearby.

**Figure 6.**
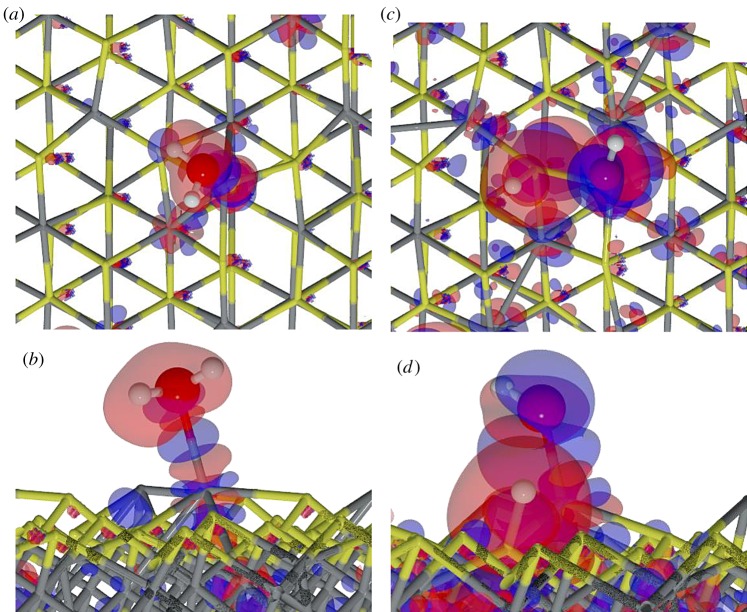
Charge flow diagrams for (*a*,*b*) molecular and (*c*,*d*) dissociativeadsorption of H_2_O on Fe_3_S_4_{111}, where red clouds show depletion and blue gain of charge density (isovalue is ±0.01 *e*^−^ Å^−3^). Grey and yellow sticks depict Fe and S, respectively; red and white balls represent O and H, respectively. (Online version in colour.)

Fe_3_S_4_{111}. The Fe_3_S_4_{111} unit cell exposes an Fe_B_ where the H_2_O molecule adsorbs exothermically (*E*_ads_=−0.56 eV). The molecule is slightly tilted with an H atom pointing towards the S sites at 2.867 Å, as shown in figure 3. As on Fe_B_-{011}, the Fe_B_ site moves perpendicularly out of the surface by 1.2 Å (*E*_def_=0.35 eV); however, the surface distortion is only local, and the sulfur bound to this Fe_B_ do not change their position significantly. The location of the Fe_B_ bonded to H_2_O corresponds to the bulk position it occupied in the pure slab before relaxation. The *E*_ads_ is practically the same as for {001}, although the Fe_B_–H_2_O distance is approximately 0.2 Åshorter, in part owing to the saturation of a dangling bond of the Fe_B_ and the formation of a surface-hydrogen bond, with a long-range contribution to the adsorption of 0.13 eV. Upon these structural changes, the H_2_O adsorption leads to a charge transfer of 0.1 *e*^−^ to the molecule and to a spin density variation of the Fe_B_ site with respect to the naked surface of 0.4 *μ*_*B*_. From the charge flow representation in figure 6*a*,*b*, we note the bond formation between the Fe and the molecule (blue cloud), as well as the polarization of the sulfur under the H and the electron relocation of the nearby metallic centres owing to the Fe_B_ geometry change.

For the dissociative adsorption process, we placed H on the S site between Fe_B_ and Fe_A_ where the H from H_2_O pointed in the molecular adsorption structure. Upon optimization, the Fe_B_–OH distance is 0.35 Åshorter than for the H_2_O molecule, which causes the Fe_B_ and the S site to move outwards with a surface destabilization of 0.54 eV. The dissociative adsorption is an unlikely process as it is 0.11 eV higher in energy than the isolated system, despite an increment in the long-range interaction of 0.10 eV. The dissociative process is accompanied by a charge transfer to the OH of 0.7 *e*^−^ and to the H ad-atom of 0.5 *e*^−^, besides a strong repositioning of the charge density (figure 6*b*). This electron rearrangement modifies the Fe_A_ charge and spin density by 0.2 *e*^−^ and 0.3 *μ*_*B*_, respectively. The dissociation mechanism has an energy barrier of 0.94 eV above the pre-adsorbed molecule that also makes the process kinetically unlikely.

### (c) Two H_2_O on Fe_3_S_4_ surfaces

**Figure 7.**
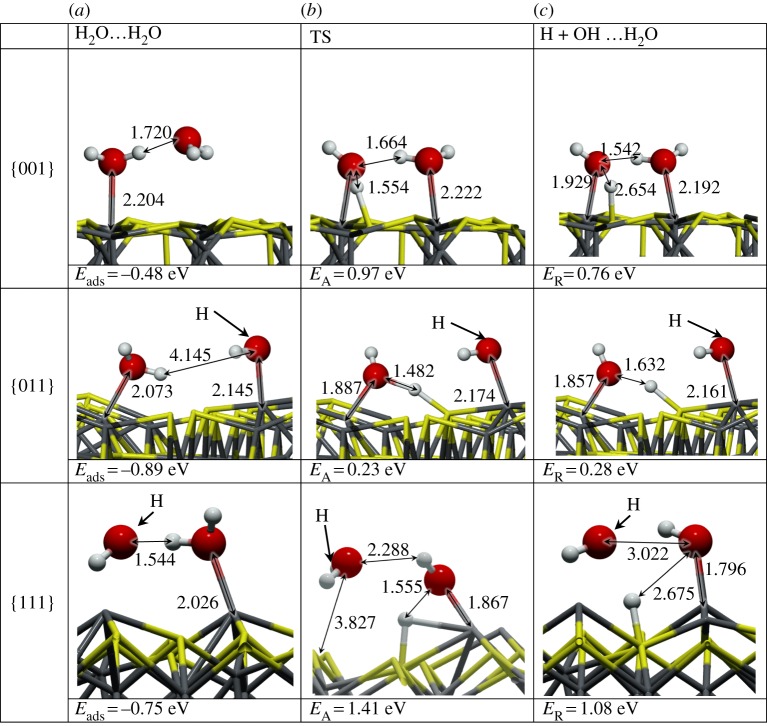
Schematic representation of H_2_O⋯H_2_O (*a*), transition state (*b*) and *H*+*OH*⋯H_2_O (*c*)on {001},{011} and {111} Fe_3_S_4_ surfaces. Grey and yellow sticks depict Fe and S, respectively; red and white balls represent O and H, respectively. The arrows indicate the position of the hidden hydrogen and all distances are in Å. (Online version in colour.)

We next studied the process of water dissociation in the presence of a nearby pre-adsorbed H_2_O molecule on the Fe_3_S_4_{001},{011} and {111} surfaces. Reactants, TSs and products are depicted in figure 7.

**Figure 8.**
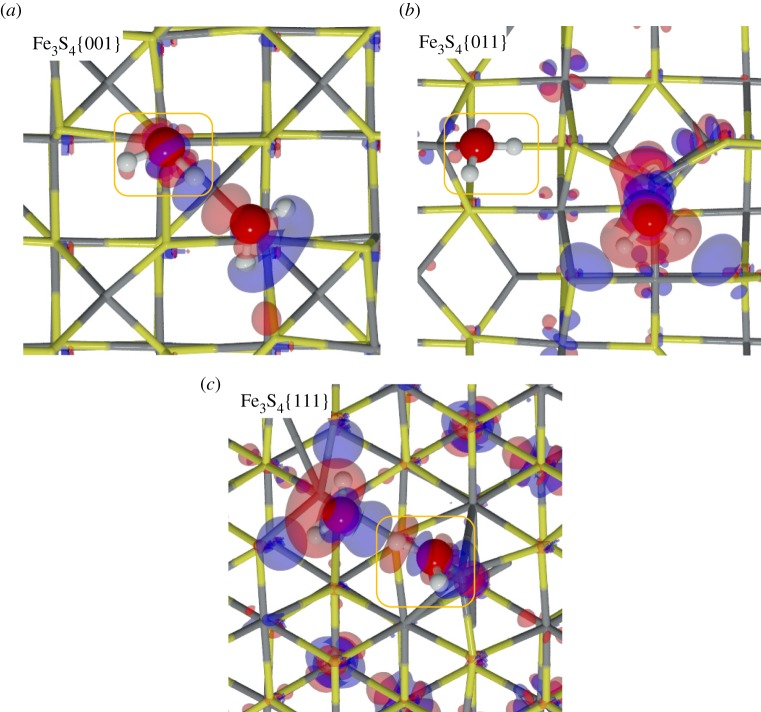
Charge flow diagrams for the interaction of a second H_2_O molecule near the pre-adsorbed molecule on Fe_3_S_4_{001}(*a*),{011} (*b*) and {111} (*c*) surfaces. Red clouds show the electron charge depletion and bluethe gain (isovalue is ±0.01 *e*^−^ Å^−3^). Grey and yellow sticks depict Fe and S, respectively; red and white balls represent O and H, respectively. The frame marks the pre-adsorbed H_2_O molecule. (Online version in colour.)

Fe_3_S_4_{001}. We brought a second H_2_O molecule close to the previously optimized system on the Fe_3_S_4_{001} but it did not adsorb on either the nearby Fe_B_ or the nearby Fe_A_. Upon optimization, the second molecule's oxygen interacts with the pre-adsorbed H_2_O via an H bond, which provides 0.49 eV to the system stabilization, with a contribution from the long-range interaction of 0.20 eV. The second molecule remains at approximately 3 Åfrom the surface and at 1.720 Åto the pre-adsorbed molecule. This interaction enlarges the H–O* distance (we use an asterisk to denote the pre-adsorbed molecule) by 0.03 Åbut H_2_O* also moves closer to Fe_B_ by 0.12 Å. The presence of the second molecule strains the angle of the pre-adsorbed molecule by 1.3° (all parameters are summarized in table 4). The electronic structure is mostly unaffected and only the bound Fe_B_ site decreases its spin density by 0.3 *μ*_*B*_. From the density flux representation in figure 8*a*, we note that S polarization appears towards the second molecule H (*d*_*S*–*H*_=2.834 Å), whereas the previous S polarization has disappeared.

**Table 4.** Adsorption energy per molecule with (and without) dispersion correction (*E*_ads_), the interaction energy of the second H_2_O molecule (Δ*E*) and distances (*d*) for the first H_2_O adsorption (denoted with asterisk) on the Fe_3_S_4_{001},{011} and {111} surfaces in the presence of a second H_2_O molecule. Gas phase H_2_O molecule has *d*_O–H_=0.971 Å.

site	*E*_ads_ (eV)	Δ*E* (eV)	*d*_O*-Fe_ (Å)	*d*_O*–H*_ (Å)	*d*_H_2_O*⋯H–OH_ (ÅA)
Fe_B_-(001)	−0.48 (−0.12)	−0.49	2.204	0.988	1.720
Fe_B,*A*_-(011)	−0.89 (−0.55)	−0.76	2.109^*a*^	0.992	4.145
Fe_B_-(111)	−0.75 (−0.33)	−0.87	2.026	1.003	1.544

^*a*^The average distance between Fe_A_, Fe_B_ and adsorbed waters.

The dissociative adsorption on the {001} surface, in the presence of a second H_2_O molecule, is only 0.05 eV more favourable than for a single molecule. The OH group is almost perpendicular to the surface (<_S–Fe–O_ =84.5°). The HO–Fe distance is 1.929 Å, which is 2.85% longer than in a single dissociated molecule despite an extra 0.1 *e*^−^ being transferred from the surface. Upon H_2_O dissociation, contiguous Fe_B_ are slightly reduced owing to the rearrangement of the electronic structure of the hydrogenated S. It makes this Fe_B_ site more likely to bind an extra H_2_O molecule, thus promoting a synergistic adsorption of a second molecule. The second molecule of water binds to the nearby active centre at an Fe–O distance of 2.192  Å, which is approximately 1 Å closer than when the molecule hovers above the non-active Fe_B_. As shown in the schematic representation of figure 7, both molecules interact through an H-bond (*d*_H…O_=1.542 Å), providing stabilization to the system. Considering the dissociation as a process that takes place on the surface and not owing to the adsorption itself, the activation energy is 0.1 eV higher than for a single molecule. This change in the energy barrier may be related to the loss of degrees of freedom owing to the second molecule binding to the surface.

Fe_3_S_4_{011}. On this surface, a second H_2_O molecule adsorbs at a low-coordinated metallic centre: Fe_B_ or Fe_A_. As a result, the two adsorbed molecules are 5 Å apart (measured between oxygens), and the interaction energy between them is negligible, *E*_coh_=0.02 eV, although the long-range contribution to the whole system's stability increases by 0.16 eV. The binding energy per molecule decreased by 0.15 eV, in agreement with an H_2_O–Fe distance of approximately 2.1 Å, leaving the S–H at 2.034 Å. We analysed the changes in the electronic structure and found that, as for the single molecule adsorption, there is no charge transfer to the molecules and only slight variations in the spin density of 0.1 *μ*_*B*_. The charge density relocation is depicted in figure 8*b*, which shows the polarization of the surface S towards the second molecule's H, whereas the structure around the pre-adsorbed H_2_O* remains unaltered.

The presence of a second H_2_O molecule, nevertheless, enhances the dissociative adsorption process by 0.04 eV compared with an isolated molecule, although the process is still less favourable than the molecular adsorption. Upon dissociation, the HO–Fe_B_ distance is 1.857 Å, which is 0.13 Åshorter than in a single dissociated molecule. Note that this is the opposite behaviour to that seen on the {001}, because both molecules adsorb strongly on the surface. The OH group remains tilted towards the H ad-atom at 1.632 Å, and there is no change in the main electronic charge. The second water molecule remains on the Fe_A_ at 2.161 Å, which is 0.04 Å closer than an isolated H_2_O on Fe_A_. As shown in the schematic representation of figure 7, the second molecule rotates approximately 120° on its own axis but it does not generate any change in the electronic structure or in the surface. The activation energy for the molecule's dissociation is approximately 0.1 eV lower than for the first molecule, thus becoming kinetically as well as thermodynamically less restricted.

Fe_3_S_4_{111}. The Fe_3_S_4_{111} surface only presents one Fe_B_ in the uppermost atomic layer where a single H_2_O molecule adsorbs. A second molecule may adsorb on Fe_A_ but this is 4.42 Åfrom Fe_B_ and, instead, the second molecule prefers to hover close to the pre-adsorbed one on Fe_B_. We found a stable state where there is an H bond between both molecules, *d*_*O*…*H*_=1.544 Å, in agreement with previous results on similar surfaces [41]. The hovering H_2_O also stabilizes the system, *E*_coh_=0.06 eV, with a total van der Waals contribution of 0.2 eV stronger than for the single H_2_O system. It also modifies the geometry of the pre-adsorbed molecule: the Fe–O* bond becomes stronger by 0.19 eV and shorter, *d*_*Fe*–O*_=2.026 Å, whereas the S–H distance of the H_2_O–Fe_B_ is also 0.06 Ålonger. Apart from the geometrical relaxation, atomic charges and spins remain as for a single molecule adsorption. From the flux diagram in figure 8*c*, the increased strength of the Fe–O* bond is shown by the increase of charge density, whereas the polarization of the H_2_O molecules causes the H_2_O lone pairs to interact with HO*–H^*δ*+^, which results in stronger polarization of the sulfur close to the second physisorbed molecule. Furthermore, figure 8*c* shows a hexagonal pattern of the charge density reallocation around the second nearest neighbours of Fe_B_, without charge or spin density transfer.

We also analysed the effect of the second water on a previously dissociated H_2_O and found that the system is 0.54 eV less stable than for molecular adsorption. Furthermore, the dissociation of the pre-adsorbed molecule has a higher energy barrier, *E*_*A*_=1.41 eV, which makes the process unlikely to occur. The hydroxy group remains on the Fe_B_ at 1.796 Åand perpendicular to the surface, binding the hovering water via an H bond (*d*_*O*…HO*_=3.022 Å) and receiving 0.2 *e*^−^ from the Fe_B_(0.14 *e*^−^) and surrounding metals through the Fe–S bond, compared with the molecular adsorbed species. Furthermore, the adsorption centre also changes its spin density in the same way as it was modified by the OH from a single molecule interaction, 0.3 *μ*_*B*_ higher than in the naked surface. The second molecule also interacts with the thiol's hydrogen at a distance of 1.854 Å. The strong interaction and the short distance to the H on the S site may lead to desorption of a hydronium cation, e.g. in solution (*H*_3_*O*^+^), and HO–Fe_B_ on the surface.

### (d) Trends and discussion

We analysed the trends in any changes in the geometry and electronic structure upon adsorption or dissociation of one and two molecules of water on the greigite surfaces. The most favourable adsorption site among the {001},{011} and {111} surfaces is the Fe_B_ (and Fe_A_ on the {011} surface). Fe_A_, located slightly underneath the S layer, is protected by the electronic cloud surrounding the S, which repulses the lone pair of electrons of the water molecule. The molecular adsorption energies are −0.35,−1.04,−1.11 and −0.56 eV on the {001}, Fe_A_-{011}, Fe_B_-{011} and {111}, respectively. These are slightly weaker than on pyrite [78,79] but quite similar to its isomorphic oxide (*E*_ads_=−0.4 to −1.0 eV) [39–41] and to FeNi_2_S_4_ surfaces [21].

**Figure 9.**
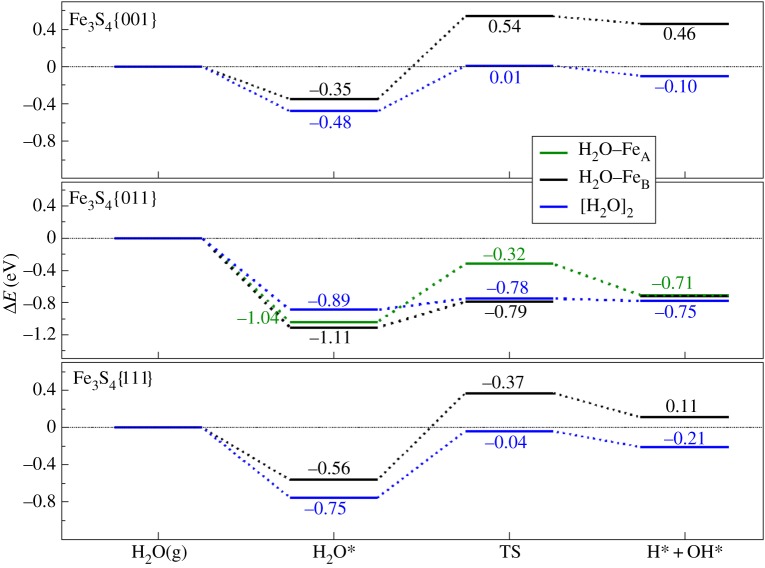
Potential energy surface for the water dissociationof the mono- (black (Fe_B_) and green (Fe_A_)) and di- (blue) molecular systems on the {001}, {011} and {111} greigite surfaces. Dotted horizontal lines show the reference energy of the isolated H_2_O and surfaces. (Online version in colour.)

The H_2_O adsorption on the surface leads to a small rearrangement of the spin densities, although with negligible charge transfer to the molecule, whereby nearby sulfur atoms polarize their electronic clouds towards both hydrogen atoms of the water molecule. Electronic structure analysis showed electron accumulation between the surface and the water molecule, indicating a chemical bond between the species. The O–H bond of the molecule may become stretched towards the polarized sulfur on the surface until its dissociation, leading to both hydroxy and thiol groups. However, the dissociation process on the different surfaces has energy barriers of approximately 0.9 eV. These energies are almost twice the main activation energies on reported Fe_3_O_4_ (*E*_*A*_<0.5 eV) [39–41]. The energy profile in figure 9 summarizes the thermodynamic unviability of the dissociation process on these surfaces. If, however, the molecule were to be dissociated, the –OH group subtracts approximately 0.7 *e*^−^ from nearby metal centres and an −SH bond is formed with a covalent character, deduced from both Bader analysis of the charges and charge density flux diagrams; similar charge-transfer characteristics have been reported for water on the pyrite {100} surface, but with water gaining a charge of only 0.03 *e*^−^, reflecting the greater stability of pyrite and its inability to be further oxidized [78]. For dissociative adsorption, the electron density is concentrated on the oxygen atom of the OH group.

**Figure 10.**
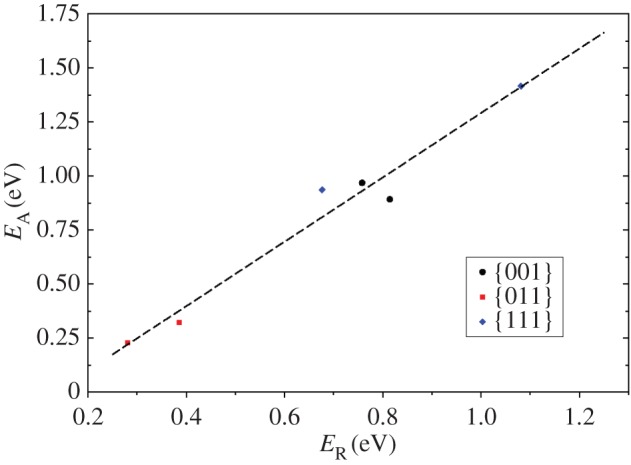
Linear relationship between the reaction and activation energies for H_2_O dissociation on the {001}, B-{011} and {111} greigite surfaces. Trend line: *E*_*A*_=−0.199+1.490⋅*E*_*R*_; *R*=0.98. (Online version in colour.)

We calculated the interaction energies of both molecules with the surface to be −0.48,−0.89 and −0.75 eV for the {001},{011} and {111}, respectively. These energies are higher than the H_2_O dimer (−0.22 eV) [80] and would lead to preferential adsorption on the surface, rather than remaining as a gas phase species. We have represented in figure 10 the activation energy as a function of the reaction energy and found a linear trend which is typical of a Brønsted–Evans–Polanyi relationship [81].

As is found for metallic or Fe_3_O_4_ surfaces [39,82], the hovering molecule on greigite remains at approximately 3 Åabove the surface, stretching the H–OH bond of the previously adsorbed molecule by approximately 0.02 Å, whereas the initial Fe–O becomes shorter by approximately 0.07 Å compared with the monomer. Even with a second molecule present, the molecular dissociation is thermodynamically unfavourable with respect to molecular co-adsorption by 0.76,0.28 and 1.08 eV for {001},{011} and {111}, respectively. The kinetics are also unfavourable towards dissociation, with activation barriers of 0.97 and 1.41 eV above the molecular state, respectively, for {001} and {111}, although for {011} the water dissociation can be achieved fairly easily (0.23 eV). Nevertheless, the combination of OH and H leading to H_2_O is both thermodynamically and kinetically favourable on the three surfaces explored.

## 4. Conclusion

We studied the iron thio-spinel Fe_3_S_4_ surfaces of the same Miller indices as the most common surfaces present in its oxide analogue magnetite, i.e. {001},{011} and {111}. All the calculations were performed by density functional theory with the Hubbard approximation (DFT+*U*), including the long-range dispersion forces as derived from the atomic polarizability. The most stable surface is the {001}, followed by {111} and {011}, in agreement with crystals from solvothermal synthesis [16]. Before relaxation, the Fe_3_S_4_{001} and {111} showed protruding Fe_A_ (and a Fe_B_ in the {111}), but these surfaces showed a greater flexibility than their oxide counterparts. The sulfide allows movements perpendicularly to the surface, causing the Fe originally above the surface plane to penetrate beneath the top atomic layer, thereby decreasing the surface energy. Similarly to corundum-structured materials [17], on the greigite surfaces the cations relaxed inwards, moving just beneath the anionic layer. However, their position is restored by water adsorption, because the binding of the molecule decreases the dangling bonds of the surface cation, thereby removing the electrostatic driving force for relaxation.

We considered both molecular and dissociative adsorption, as well as the dissociation of the water molecule once adsorbed on the surface. We have concluded that the water dissociation is thermodynamically unfavourable. In contrast, H_2_O formation has a TS at less than 0.5 eV, indicating a fast recombination. We have also provided insights into the synergistic adsorption of the second H_2_O molecule on a nearby HO–Fe group on the Fe_3_S_4_{001} owing to the electronic structure relocation. Similar synergistic adsorption has been observed on violarite [21], as well as in the adsorption of an ethanol and an ethanol–water dimer to Rh(111) [16,83,84]. Greigite does not show the same affinity towards dissociation of water as does Fe_3_O_4_ [36], and molecular adsorption is the most favourable mode on the sulfide. This difference is due to the more acidic character of the –SH group compared with the –OH of the oxide. Therefore, to dissociate water and adsorb H ad-atoms on Fe_3_S_4_ surfaces for the reduction processes, a source of potential is required such as a natural chemiosmotic or external applied potential.

## Supplementary Material

Surface properties, Simulated STM and Morphology

## Data accessibility

The datasets supporting this article have been uploaded as part of the supplementary material. Electronic supplementary material contains details of all surface terminations, such as interatomic distances, local and per unit formula magnetization, work function and surface energy. It also provides simulated STM and Wulff construction figures. All data created during this research are openly available from the University of Cardiff Research Portal at http://dx.doi.org/10.17035/d.2016.0008383220.
