# Supplementary material for: Catalytic water dissociation by greigite Fe3S4 surfaces: density functional theory study
Source: Proc Math Phys Eng Sci. 2016 Apr;472(2188):20160080. doi: 10.1098/rspa.2016.0080 (PMC4892285; doi:10.1098/rspa.2016.0080)
Supplement: Surface properties, Simulated STM and Morphology [file rspa20160080supp1.doc]

**Electronic Supplementary Material**

**Catalytic Water Dissociation by Greigite Fe_3_S_4_ Surfaces: DFT Study**

A. Roldan*, and N.H. de Leeuw

This file contents a compilation of figures and tables that complement the results and discussion in the publication with title “**A Density Functional Theory Study of Water Adsorption and Dissociation on Greigite Fe_3_S_4_ Surfaces**”.

Content:

- [PRISTINE SURFACE](#__RefHeading___Toc442198928)

[Surface properties](#__RefHeading___Toc442198929)

[Simulated STM and Morphology](#__RefHeading___Toc442198930)

- [References](#__RefHeading___Toc442198931)

# PRISTINE SURFACE

## Surface properties

Atomic surface relaxation slightly changes and stabilises the loss of coordination decreasing the surface energy (γ), defined by **equation S1**. We express the extent of surface energy change by a percentage (**equation** **S2**). In these equations, *E_slab_* is the absolute energy of the slab with one relaxed side, *E_slab_^unrelaxed^* is the absolute energy of the unrelaxed slab, *n* is 1 as there are the same number of formula units in the slab as in the bulk cell, *E_Bulk_* is the bulk energy and *A* is the area exposed by the surface.

$\gamma=\frac{E_{slab}^{relaxed}-nE_{Bulk}}{A}-\frac{E_{slab}^{unrelaxed}-nE_{Bulk}}{2A}$ (**Eq. S1**)

$\%relaxation=\frac{\gamma_{unrelaxed}-\gamma_{relaxed}}{\gamma_{unrelaxed}}\times100$ (**Eq. S2**)

**Table SI**. Surface energy (γ) values for different terminations before and after structural relaxation of the prominent Fe_3_S_4_ surfaces. Although {011} has two terminations, we have provided the average surface energy, as the surfaces are complementary but not symmetric.

|  |  | γ-unrelaxed (J m^-2^) | γ-relaxed (J m^-2^) | Relaxation (%) |
| --- | --- | --- | --- | --- |
| {001} | Type-A | 1.1 | 0.6 | 41.0 |
|  | Type-B | 2.2 | 1.3 | 42.0 |
| {011} | | 1.5 | 1.0 | 42.9 |
| {111} | Type-A | 1.2 | 0.8 | 35.3 |
|  | Type-B | 1.9 | 0.9 | 52.8 |

We next discuss the surface reconstruction for the three different surfaces and the most stable terminations where the interatomic distances change to a greater or lesser extent, depending on the kind of surface. In the {001}-A surface, the Fe_A_ atoms move 0.30 Å towards the bulk, increasing the main d(Fe_A_-S) by ~0.08 Å with respect to the bulk distances, while the d(Fe_B_-S) remains around 2.39 Å. In the relaxation process, the uppermost Fe_A_ move down below the Fe_B_-S top-layer to form a neighbouring pair with already present Fe_A_, which stabilizes the surface by ~40%. During the {011}-A surface relaxation, cations move by up to 0.42 Å for Fe_A_ and 0.34 Å for Fe_B_, modifying their distance to sulphur by 0.08 and 0.04 Å respectively. Again, the large Fe_A_ relaxation moves the cations towards the bulk, placing them at the same level as the top S atomic layer. The atomic contraction on the {111}-A surface is even larger than for {001}-A: Fe_A_ and Fe_B_ move down to the bulk by 0.75 and 0.24 Å respectively. The distance d(Fe_A_-S) increases by 0.11 Å to 2.28 Å and d(Fe_B_-S) by 0.03 Å to 2.40 Å with respect to the bulk distances.

The size of synthetized particles may be crucial for their electronic properties. In some systems containing ultrafine magnetic particles, the spins on external atomic layers can be inclined at various angles with respect to the directions of the net moment, modifying the total magnetic moment, M_s_.[^1^](#_ENREF_1) Because of surface optimization, atomic charges slightly increase on Fe_A_ and decrease on Fe_B_ by less than 0.1 e in both cases. Otherwise, the measure of the spin moment is more sensitive to structural changes showing a spin variation on both kinds of Fe (note the negative values) and therefore a different M_s_ per unit formula compared with bulk analysis. For the {001} surface, both spins on Fe slightly increase by 0.1 μ_B_, but this is not the case for {011}, which remains as in the bulk structure, or the {111} surface, where the atomic spin orientation decreases. The surface effect on the magnetic moment may be observed and measured on ultrathin nanoparticles. At these sizes, there are more atoms in the surface than in the bulk and the relative weight of the surface M_s_ becomes important. In contrast, for larger particles, the surface M_s_ becomes irrelevant compared to the bulk M_s_. The electronic structure is also expressed by the work function (Φ) that indicates the energy required to move one electron from the Fermi level to the vacuum. Among the surfaces reported in this paper, the lowest Φ is found on the Fe_3_S_4_ B-{011} (4.92 eV), probably due the presence of octahedral ferrous-ferric atoms in the uppermost layer containing the electrons at the highest occupied band.

Below, we show schematic representations and some important properties of the greigite surfaces, taking into account the possible terminations of the uppermost atomic layer. Grey balls and sticks denote Fe atoms and dark-yellow the S anions in the initial and final side view structure. Properties are denoted as follows: d is the main distance between the same kind of atoms and its first-neighbours, q is the main atomic Bader charge, m_s_ is the main magnetization, the global magnetization of saturation per unit formula is M_s_, Φ is the work function of the relevant surface and γ is the surface energy.

**Figure S1**. Surface properties and schematic representation of the side view for the different termination of {001}, {011} and {111} Fe_3_S_4_ surfaces.

| **Fe_3_S_4_{001} -A** | |  |  |
| --- | --- | --- | --- |
| Area /Å^2^ | 93.5 |
| d(Fe_Td_-S) /Å | 2.256 | 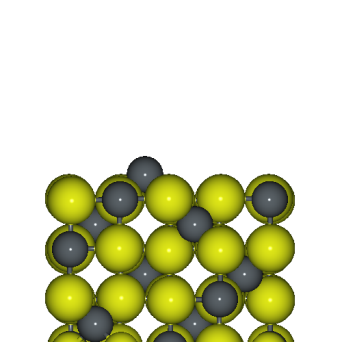 | 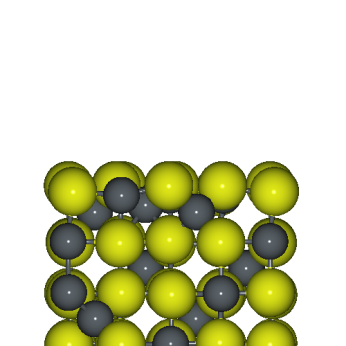 |
| d(Fe_Oh_-S) /Å | 2.392 |
| q(Fe_Td_) /e | 1.11 |
| q(Fe_Oh_) /e | 1.13 |
| q(S) /e | -0.84 |
| m_s_(Fe_Td_) /μ_B_ | 2.93 |
| m_s_(Fe_Oh_) / μ_B_ | 3.14 |
| M_s_ / μ_B_ u.f.^-1^ | 3.66 |
| Φ /eV | 5.13 | Initial | Final |
| γ /J m^-2^ | 0.6 | Cross section | |

| **Fe_3_S_4_{001} -B** | |  |  |
| --- | --- | --- | --- |
| Area /Å^2^ | 93.5 |
| d(Fe_Td_-S) /Å | 2.387 | 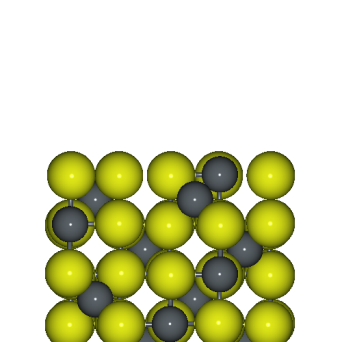 | 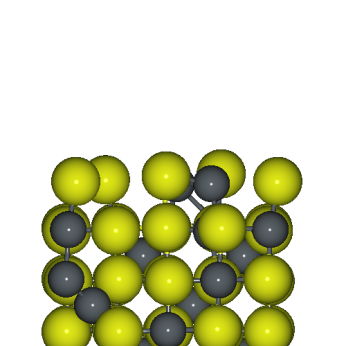 |
| d(Fe_Oh_-S) /Å | 2.435 |
| q(Fe_Td_) /e | 1.05 |
| q(Fe_Oh_) /e | 1.06 |
| q(S) /e | -0.79 |
| m_s_(Fe_Td_) /μ_B_ | 2.95 |
| m_s_(Fe_Oh_) / μ_B_ | 3.05 |
| M_s_ / μ_B_ u.f.^-1^ | 3.43 |
| Φ /eV | 5.19 | Initial | Final |
| γ /J m^-2^ | 1.3 | Cross section | |

| **Fe_3_S_4_{011} -A** | |  |  |
| --- | --- | --- | --- |
| Area /Å^2^ | 132.3 |
| d(Fe_Td_-S) /Å | 2.256 | 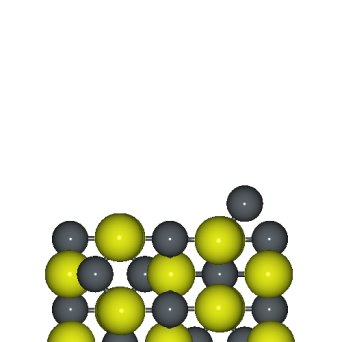 | 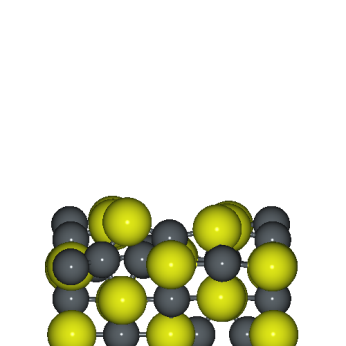 |
| d(Fe_Oh_-S) /Å | 2.405 |
| q(Fe_Td_) /e | 1.10 |
| q(Fe_Oh_) /e | 1.10 |
| q(S) /e | -0.83 |
| m_s_(Fe_Td_) /μ_B_ | 2.99 |
| m_s_(Fe_Oh_) / μ_B_ | 3.01 |
| M_s_ / μ_B_ u.f.^-1^ | 3.24 |
| Φ /eV | 5.24 | Initial | Final |
| γ /J m^-2^ | 1.0 | Cross section | |

| **Fe_3_S_4_{011} –B** | |  |  |
| --- | --- | --- | --- |
| Area /Å^2^ | 132.3 |
| d(Fe_Td_-S) /Å | 2.352 | 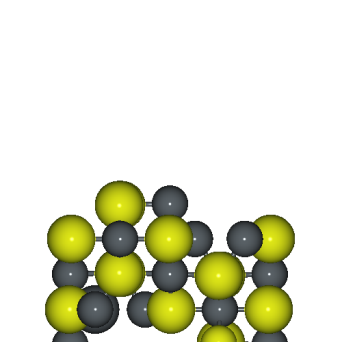 | 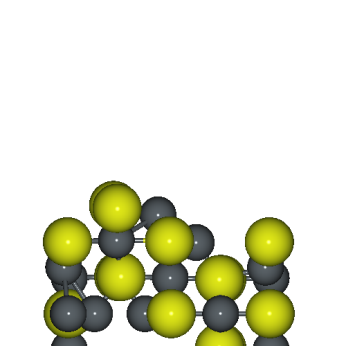 |
| d(Fe_Oh_-S) /Å | 2.448 |
| q(Fe_Td_) /e | 1.04 |
| q(Fe_Oh_) /e | 1.11 |
| q(S) /e | -0.82 |
| m_s_(Fe_Td_) /μ_B_ | 2.99 |
| m_s_(Fe_Oh_) / μ_B_ | 2.98 |
| M_s_ / μ_B_ u.f.^-1^ | 3.26 |
| Φ /eV | 4.92 | Initial | Final |
| γ /J m^-2^ | 1.0 | Cross section | |

| **Fe_3_S_4_{111} -A** | |  |  |
| --- | --- | --- | --- |
| Area /Å^2^ | 81.0 |
| d(Fe_Td_-S) /Å | 2.289 | 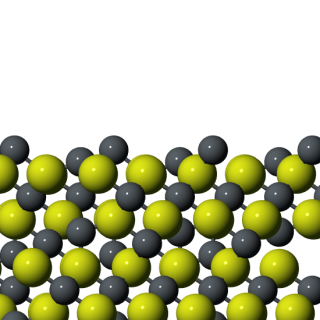 | 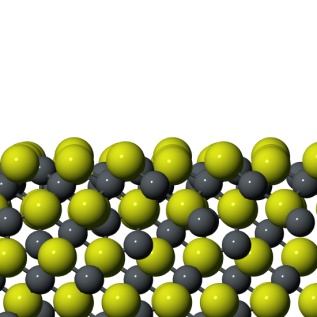 |
| d(Fe_Oh_-S) /Å | 2.399 |
| q(Fe_Td_) /e | 1.08 |
| q(Fe_Oh_) /e | 1.10 |
| q(S) /e | -0.82 |
| m_s_(Fe_Td_) /μ_B_ | 1.36 |
| m_s_(Fe_Oh_) / μ_B_ | 2.47 |
| M_s_ / μ_B_ u.f.^-1^ | 2.93 |
| Φ /eV | 5.63 | Initial | Final |
| γ /J m^-2^ | 0.8 | Cross section | |

| **Fe_3_S_4_{111} -B** | |  |  |
| --- | --- | --- | --- |
| Area /Å^2^ | 81.0 |
| d(Fe_Td_-S) /Å | 2.258 | 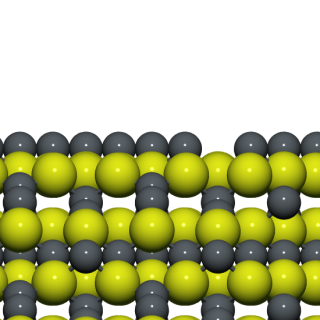 | 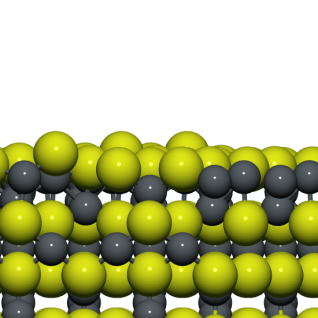 |
| d(Fe_Oh_-S) /Å | 2.404 |
| q(Fe_Td_) /e | 1.11 |
| q(Fe_Oh_) /e | 1.04 |
| q(S) /e | -0.80 |
| m_s_(Fe_Td_) /μ_B_ | 2.68 |
| m_s_(Fe_Oh_) / μ_B_ | 3.00 |
| M_s_ / μ_B_ u.f.^-1^ | 3.53 |
| Φ /eV | 5.04 | Initial | Final |
| γ /J m^-2^ | 0.9 | Cross section | |

## Simulated STM and Morphology

The scanning tunnelling microscopy (STM) images are calculated using the Tersoff-Hamann formalism,[^2^](#_ENREF_2) where the tunnelling current in an STM experiment is proportional to the local density of states (LDOS) integrated from the Fermi energy (E_F_) to the bias. According to this treatment, the tunnelling matrix element between the tip and the surface is proportional to the eigenstate of the investigated surface as a function of the tip position. In order to simulate a STM topographic image, we integrated our DFT partial charge density from E_F_ to 1 eV using the HIVE program.[^3^](#_ENREF_3) It is implemented in its most basic formulation, approximating the STM tip by an infinitely small point source. An STM in constant current mode follows an isosurface of constant integrated LDOS at certain distance above the highest atom of the surface or a constant charge level (e^−^/Å^3^). As such, this construction returns a height as a function of the virtual tip position (x, y) and it is plotted linearly onto a corresponding scale.

**Figure S2**. Modelled STM images at a constant bias of 5∙10^-4^ e/Å^3^ corresponding to a tip-surface distance of 2.350, 2.428 and 2.495 Å of the most stable surface of a 5x5 supercell of {001}, {011} and {111} Fe_3_S_4_ surfaces respectively. Dark-blue and bright-yellow indicate electron-poor and -rich respectively.

| 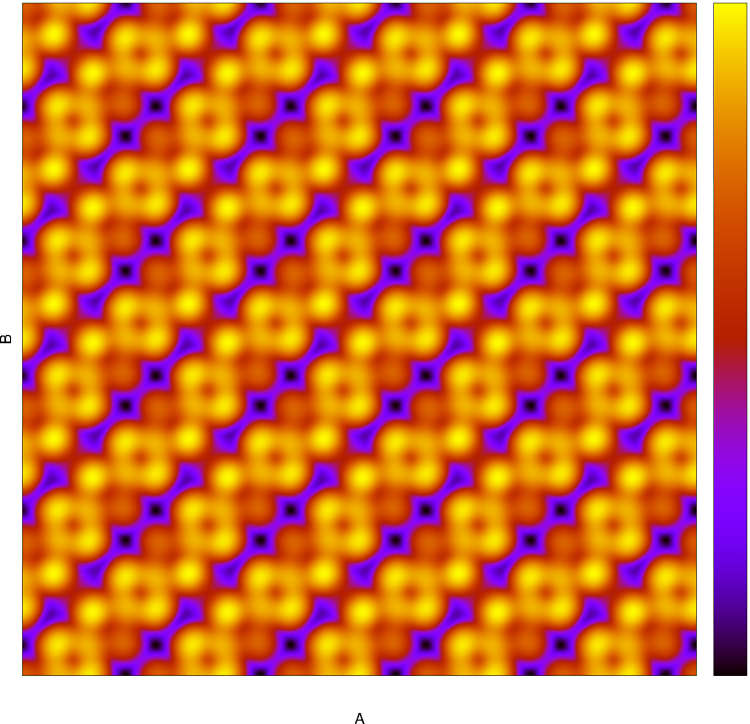  **Fe_3_S_4_{001}** | 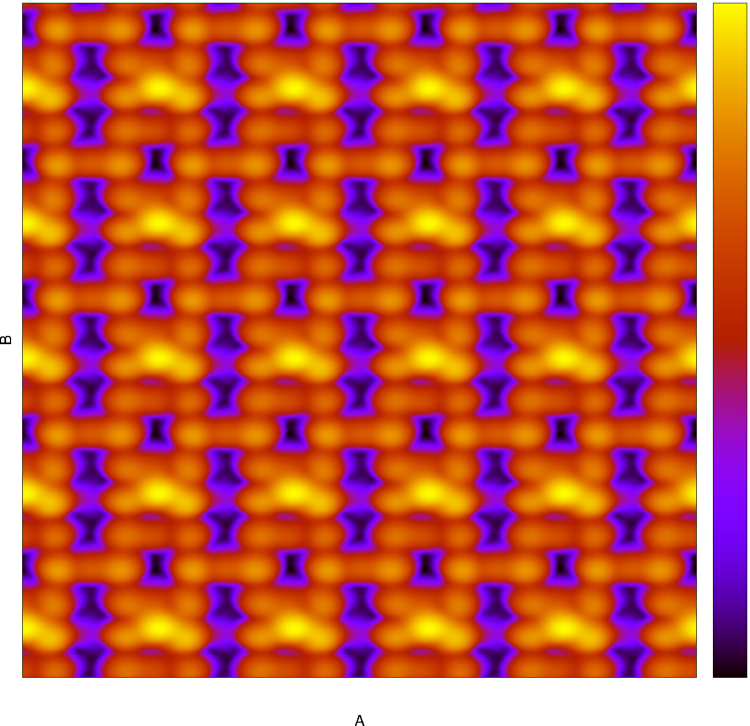  **Fe_3_S_4_{011}** |
| --- | --- |
| 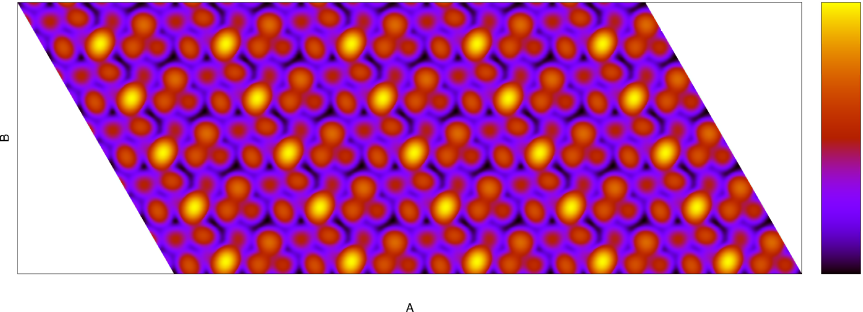  **Fe_3_S_4_{111}** | |

**Figure S3**. Wulff structure representation of a Fe_3_S_4_ moiety derived from the relative surface stability,[^4^](#_ENREF_4) axis are also indicated with arrows.


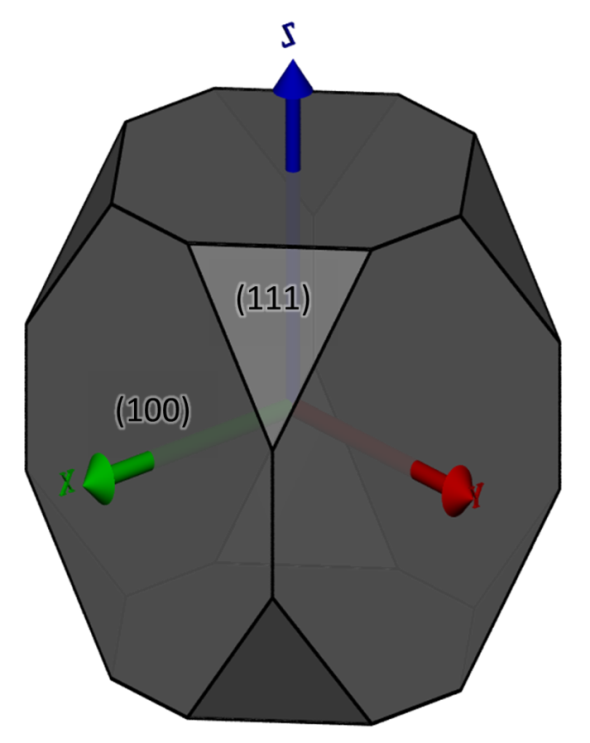


# References

1. R. H. Kodama, A. E. Berkowitz, E. J. McNiff and S. Foner, *Phys. Rev. Lett.*, 1996, **77**, 394-397.

2. J. Tersoff and D. R. Hamann, *Physical Review B*, 1985, **31**, 805-813.

3. D. E. P. Vanpoucke and G. Brocks, *Physical Review B*, 2008, **77**, 241308.

4. G. Wulff, *Zeitschrift Fur Krystallographie Und Mineralogie*, 1901, **34**, 449-530.
